# Flow patterns through vascular graft models with and without cuffs

**DOI:** 10.1371/journal.pone.0193304

**Published:** 2018-02-23

**Authors:** Chia Min Leong, Gary B. Nackman, Timothy Wei

**Affiliations:** 1 Department of Mechanical, Aerospace & Nuclear Engineering, Rensselaer Polytechnic Institute, Troy, New York, United States of America; 2 Division of Vascular Surgery, Robert Wood Johnson Medical School, New Brunswick, New Jersey, United States of America; 3 Department of Mechanical and Materials Engineering, University of Nebraska, Lincoln, Nebraska, United States of America; Coastal Carolina University, UNITED STATES

## Abstract

The shape of a bypass graft plays an important role on its efficacy. Here, we investigated flow through two vascular graft designs–with and without cuff at the anastomosis. We conducted Digital Particle Image Velocimetry (DPIV) measurements to obtain the flow field information through these vascular grafts. Two pulsatile flow waveforms corresponding to cardiac cycles during the rest and the excitation states, with 10% and without retrograde flow out the proximal end of the native artery were examined. In the absence of retrograde flow, the straight end-to-side graft showed recirculation and stagnation regions that lasted throughout the full cardiac cycle with the stagnation region more pronounced in the excitation state. The contoured end-to-side graft had stagnation region that lasted only for a portion of the cardiac cycle and was less pronounced. With 10% retrograde flow, extended stagnation regions under both rest and excitation states for both bypass grafts were eliminated. Our results show that bypass graft designers need to consider both the type of flow waveform and presence of retrograde flow when sculpting an optimal bypass graft geometry.

## II. Introduction

The efficacy of bypass grafts depends on its shape [1]. Numerous bypass grafts with cuff and patch technologies at the anastomosis like Linton patch [2], Miller cuff [3], Tyrell vein collar [4] and Taylor patch [5] had been designed to satisfy this goal. Cuffs and patches could be harvested from autologous veins or pre-formed during the manufacturing of bypass grafts. These designs having different geometries and sizes at the anastomosis would have an implication on hemodynamics in that region.

Maintaining the proper function of bypass grafts is strongly dependent on hemodynamics. Endothelial cells forming a cellular monolayer that lines the arterial walls are known to respond to hemodynamic loading in a process known as mechanotransduction [6]. Due to altered hemodynamic conditions at the anastomosis, regions of high shear stress may occur and are known to cause cell damage [7] while regions of low shear stress would lead to intimal hyperplasia, characterized by the abnormal proliferation of smooth muscle cells [8–13].

Flow patterns in the anastomosis have been extensively studied due to their importance in the design of an optimal bypass graft [14–18]. Although the effect of retrograde flow has been investigated, where a majority of the flow exits the anastomosis at the distal end, with the small remaining portion exiting proximally to supply blood to the small arterial branches at that end [16], it is sometimes neglected [15, 18]. In this study, we examined two pulsatile waveforms of rest and excitation states, with 10% and without retrograde flow (*i*.*e*. flow toward the native artery) for two different bypass graft models—a straight end-to-side (without cuff) and a contoured end-to-side (with cuff). These four flow conditions provide a coarse mapping that span a wide range of physiologic conditions characteristic of patients with bypass grafts. We used Digital Particle Image Velocimetry (DPIV) to visualize flow through these vascular grafts. The specific objectives of this study were to identify and understand differences between these two vascular graft designs, and to highlight possible clinical conditions which might arise due to these vascular graft designs.

## III. Experimental apparatus and methods

**Fig 1:**
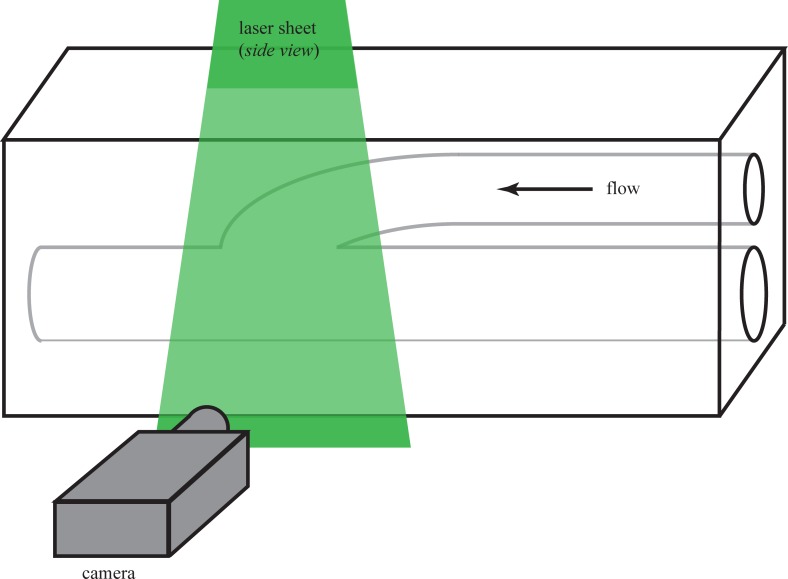
Sketch of a model vascular graft along with the experimental setup. Flow is right-to-left. The model shown is configured for DPIV measurements; the laser sheet is shown entering from the top of the page. The digital camera captures images of light scattering from small neutrally-buoyant particles seeded in the flow.

We conducted a series of Digital Particle Image Velocimetry (DPIV) experiments to visualize the differences between flow patterns through a non-cuffed versus a cuffed vascular graft model. In all cases, we conducted experiments using transparent cast models of graft geometries. A simplified sketch of the experiment is shown in Fig 1.

### III.1. Graft models

Two different graft geometries, non-cuffed (or straight end-to-side) and cuffed (or contoured end-to-side), were manufactured in rectangular, transparent, optically clear silicon (Sylgard-184, Dow Corning Corp., Midland, Michigan) blocks using a lost-material casting technique [19]. Briefly, the bypass graft models were cast using a low melting point metal (Cerrolow 117, Cerro Metal Products, Bellafonte, Pennsylvania) in an aluminum mold. These metal casts were then placed and held rigid in plastic housings before pouring silicon into the housings. After the silicone had cured, the metal casts could be easily removed by melting them. Finally, the desired bypass graft geometry remained.

The blocks were approximately 30 cm long x 10 cm high x 5 cm wide. On the upstream (proximal) end of the block there were two quick-disconnect fittings (CPC Series, Cole-Parmer, Vernon Hills, Illinois), one to allow flow into the bypass vessel and the second to permit controlled ‘leakage’ flow out of the native artery. On the downstream end, there was a single connection for outflow through the distal end of the native artery. For both the graft models, the bypass and native artery diameters were 7 mm and 5 mm, respectively, and these were measured at the inlet end of the vascular graft model.

The principal advantage of transparent silicon is the full optical access it provides. An additional benefit was the inherent degree of vessel compliance. The model walls were not fully rigid; some degree of radial expansion and contraction could be observed throughout each cardiac cycle. For this study, however, compliance effects were not examined in detail.

**Fig 2:**
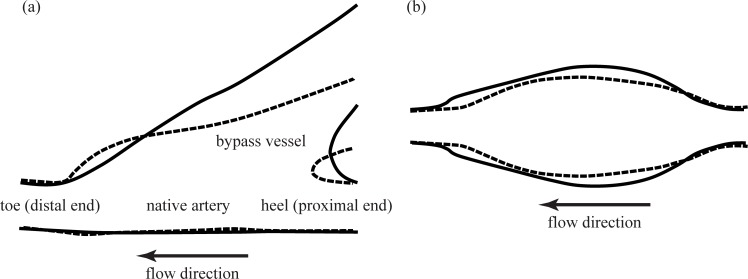
Sketches of the bypass graft models. (a) Side view and (b) bottom view. Dashed lines indicate the straight end-to-side model and solid lines indicate the contoured end-to-side model. Flow is right-to-left with the native vessel aligned horizontally across the field-of-view.

Sketches of the straight and contoured end-to-side models appear in Fig 2. For both of these models, we focused on the flow primarily on the anastomosis. Note that the straight end-to-side has a more bulbous toe region and the angle at which the graft attaches to the native vessel is smaller than for the contoured end-to-side, shown in Fig 2A. For simplicity, regions of interest will be identified relative to the side view orientation shown in Fig 2A. Thus, the flow is right-to-left. The bypass is attached to the top of the native artery and the undisturbed surface of the native vessel is the bottom.

A sense of the three-dimensionality of the geometry is provided by the corresponding bottom view images in Fig 2B. One can clearly see that the anastomosis for the contoured end-to-side is significantly more voluminous than its straight end-to-side counterpart. Flow is again right-to-left.

### III.2. Pulsatile flow pump

**Fig 3:**
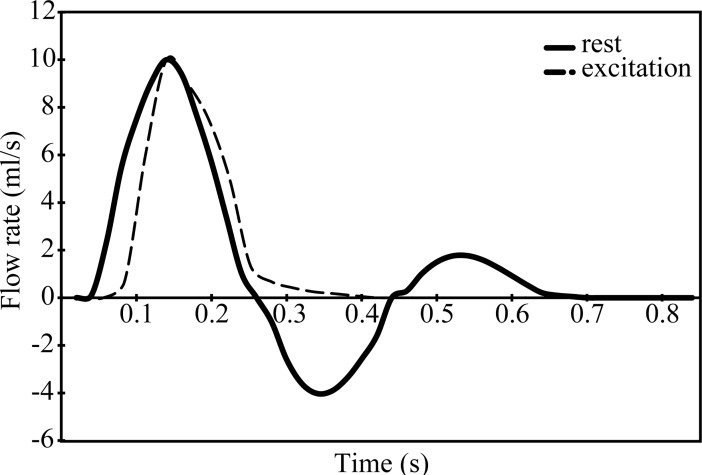
Pulsatile flow waveforms of interest corresponding to rest and excitation states.

Pulsatile flow was generated using a computer controlled pump (CompuFlow1000, Shelley Imaging Medical Technologies, Toronto, Ontario). The pump includes software to create pulsatile flows that are physiologically matched to different locations along the human vasculature. Pulsatile flow waveforms for the rest and the excitation states are shown in Fig 3. The pulse rate was approximately seventy cycles per minute. The Womersley number for both waveforms based on the radius of the bypass artery was 5. The Reynolds number based on the diameter of the bypass artery and maximum flow rate was about 500. As will be described in §III.4, we made measurements for both no leakage, or retrograde flow, (100–0) and for 10% retrograde flow through the proximal end of the native artery (90–10). The numbers, 100–0 and 90–10, refer to the relative amount of flow in percent exiting from the distal end of the native artery and the retrograde flow out the proximal end.

### III.3. Digital Particle Image Velocimetry (DPIV)

We conducted DPIV experiments [20] in this study to visualize flow through different graft models. The working fluid for these experiments was a mixture of 60% water and 40% glycerol by volume. This is a standard solution replicating the Newtonian viscosity of blood of 4 cP [21]. The working fluid is also index matched with the bypass graft models [22]. The fluid was seeded with red fluorescent polyethylene particles (UVPMS-BR, Cospheric, Santa Barbara, California). These particles were neutrally buoyant and had a nominal diameter of 70 μm. It had a maximum excitation wavelength of 575 nm and a maximum emission wavelength of 605 nm. The advantage of fluorescing particles is that it is possible to optically filter the incident laser light from the video images. Filtering was accomplished using a longpass filter with a cut-on wavelength of 600 nm. This is critical for reducing/eliminating optical noise in the form of laser light scattering from the vessel walls.

We used an in-house developed DPIV cross-correlation technique to process the DPIV images [23, 24]. This program employed a two-step correlation technique to obtain velocity. We used this technique to achieve a wide dynamic range of displacements and high spatial resolution. We used coarse and fine correlation windows of 128 x 128 pixels and 64 x 64 pixels, respectively. There correlation windows were overlapped by 75%.

### III.4. Data acquisition and flow conditions

Two graft geometries, straight end-to-side and contoured end-to-side, were each studied under the four different flow conditions as shown in Table 1. As noted in §III.2., flow with (90–10) and without (100–0) retrograde flow out the proximal end (*i*.*e*. right side) of the native artery was examined. This was done for cardiac cycles corresponding to the rest and the excitation states.

**Table 1:** Cases studied for two graft geometries (*i*.*e*. straight end-to-side and contoured end-to-side).

	Resting Cardiac Cycle	Excitation Cardiac Cycle
**100–0** **(No Retrograde Flow)**	**X**	**X**
**90–10** **(10% Retrograde Flow)**	**X**	**X**

For each case, DPIV measurements were made from the side, Fig 2A, and below, Fig 2B. The bottom view measurement was done by simply laying the model on its side so the camera looked in through the bottom face. Thus, a total of eight sets of DPIV data were taken for each model; sixteen measurements were made in all.

## IV. Results

For all DPIV data presented in this section, velocity vectors are superimposed on outline of the graft models. The magnitude and direction of the flow at any point in the field of view is indicated by the length and orientation of the velocity vectors, respectively. In addition, vector colors indicate local vorticity at each point of the flow. While vorticity is a measure of fluid rotation, at the graft boundaries, vorticity is also proportional to wall shear stress. In light of this, red velocity vectors correspond to regions of high counter-clockwise shear. Blue vectors correspond to regions of high clockwise shear. Regions of zero shear appear with green vectors. Note that it is possible to have high *speed* regions with low shear (*e*.*g*. uniform flow); color should *not* be confused with speed. Identical color spectra and velocity length scales were used for every vector field acquired in this study. It is therefore possible to directly compare color and vector length between the different cases presented in the following sections.

Specific flow phenomena to watch for include:-

- separation from the bypass vessel walls approaching the anastomosis,
- resulting formation of recirculation zones both at the toe and bottom of the anastomosis,
- presence or absence of stationary stagnation points in the anastomosis.

### IV.1. Comparison of the two geometries without retrograde flow (100–0 split)

#### IV.1a The rest case

**Fig 4:**
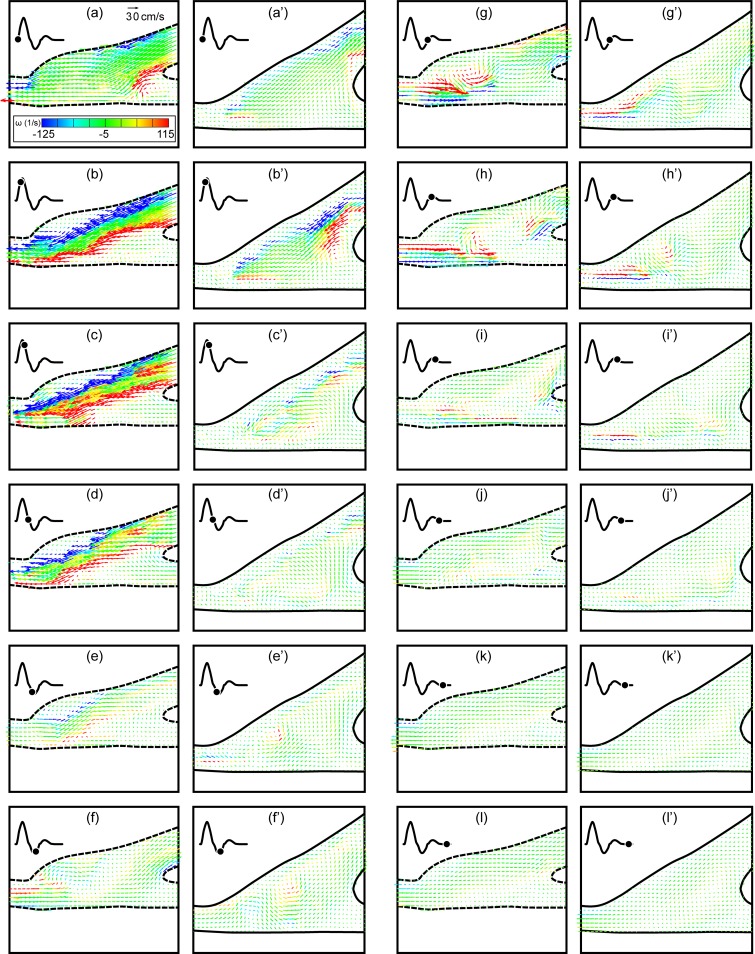
Direct comparison of the straight end-to-side (a-l) and the contoured end-to-side (a’-l’) grafts over one rest cardiac cycle. Time between successive vector fields is 70 msec. Contour legend and reference vector is shown in (a).

The first comparison is between the straight end-to-side and the contoured end-to-side grafts with no retrograde flow and with a cardiac cycle corresponding to an adult in a rest state. Side-to-side comparisons for a complete cardiac cycle are shown in Fig 4. Twelve consecutive DPIV vector fields corresponding to one complete cardiac cycle are shown for each model in two columns. The time between images is 70 msec and begins at the start of the cardiac cycle. Straight end-to-side vector fields are labeled a-l while corresponding contoured end-to-side data are labeled a’-l’.

One can immediately see distinct differences between the straight end-to-side and the contoured end-to-side grafts. Velocities are significantly larger in the straight end-to-side during systole. Shear stresses are also much higher (more red and blue) and more variable. Onset of separation and development of a recirculation zone in the cuff region of the straight end-to-side, is clearly visible in Fig 4B–4D. Note that the reattachment point at the toe of the graft experiences high oscillatory shear stresses.

It is also noteworthy that flow along the bottom wall of the native artery in the straight end-to-side, beneath the heel of the graft, remains essentially stagnant for most of the cardiac cycle. This can be problematic because fluid trapped in this stagnation region may mean a lack of waste removal and/or oxygen and nutrients transport to the underlying endothelial cells. Stagnation region has been previously determined to correspond to the common sites of intimal hyperplasia [25, 26].

Flow in the contoured end-to-side, by comparison, is more benign. Stress levels and velocity magnitudes are lower throughout the anastomosis. In addition, the flow is more laminar and more stable. Strong eddies do not roll up the way they do in the straight end-to-side. This is seen by comparing Fig 4G with 4G’, or 4H with 4H’. Every part of the anastomosis of the contoured end-to-side, however, experiences flow for a significant fraction of the cardiac cycle. As a result, the underlying cells are more likely to be subjected to healthy pulsatile stresses and regularly supplied with fresh nutrients.

**Fig 5:**
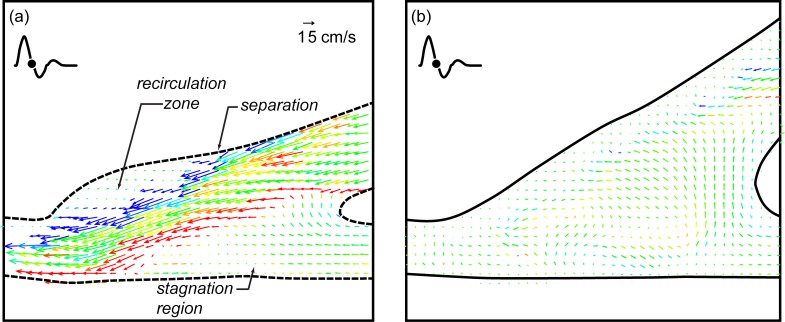
Close up of Fig 4D and 4D’. Separation, recirculation and stagnation are clearly visible in the straight end-to-side (a) while similar phenomena are weaker or nonexistent in the contoured end-to-side (b).

A close-up of Fig 4D and 4D’ are shown in Fig 5A for the straight end-to-side graft and 5b for the contoured end-to-side graft. Again the straight end-to-side is on the left and the contoured end-to-side model is on the right. Flow separation upstream of the bulge in the straight end-to-side cuff, the resulting recirculation zone and the surface along which flow remains stagnant for much of the cardiac cycle are labeled in Fig 5A. Note that these features are either non-existent or significantly weaker in the contoured end-to-side model.

**Fig 6:**
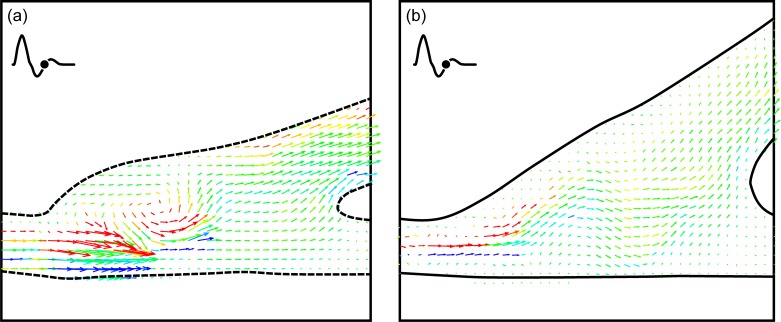
Comparison of back flow unsteadiness occurring during diastole for the rest case. Flow in the straight end-to-side rolls up into a strong vortex. Note that there is some flow through the stagnation region (indicated in Fig 5A), but this is in the counter-flow direction.

Close ups of Fig 4G and 4G’ appear in Fig 6A and 6B. These vector fields were acquired during diastole. In the rest case, there is some back flow up the bypass graft. This is visible in both vector fields. The salient feature of Fig 6 is the high degree of flow irregularity in the straight end-to-side model; the back flow rolls up into a strong vortex below the toe of the graft. Where there is some waviness in the contoured end-to-side graft, the disturbance is not as energetic as in the straight end-to-side graft.

It is worth noting that the backflow associated with peak diastole does disrupt the stagnation zone for ~0.2 sec or 24% of the cardiac cycle. The ‘refreshment’ of the endothelium below the stagnation zone is, however, a reverse flow.

#### IV.1b The excitation case

**Fig 7:**
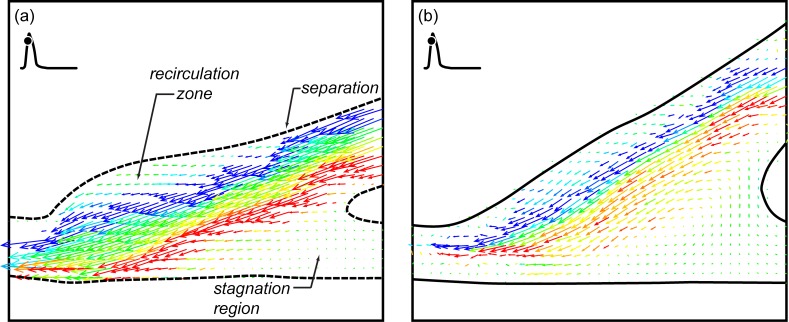
Side-by-side comparison of the straight and contoured end-to-side grafts near peak systole with an excitation cardiac cycle. Note the stagnation region is larger and more stable than in the rest case.

The stagnation region in the straight end-to-side graft is much more pronounced when the cardiac cycle is in the excitation state. A side-to-side comparison of flow near peak systole is shown in Fig 7. Again, the straight end-to-side model appears on the left and key flow features are labeled. While there are brief periods in the rest cardiac cycle in which there is flow through the area labeled as the stagnation region, this does not appear to happen during excitation. This is presumably because the momentum of the bypass jet is much larger during excitation than in rest. Fluid travels along the bypass into the native artery with little recirculation in the anastomosis.

Fig 7B reveals a stagnation region in the contoured end-to-side model, analogous to that discussed for the straight end-to-side model, Fig 7A. While this is a stagnation region, the key difference is that, for contoured end-to-side model, such stagnation regions are short lived. Using the time sequence in Fig 4 as a representative example, it can be seen that stagnation regions appear at a given location for only one or two consecutive vector fields. For the contoured end-to-side model, this may imply that the entire endothelium is perpetually supplied with ‘fresh’ blood.

**Fig 8:**
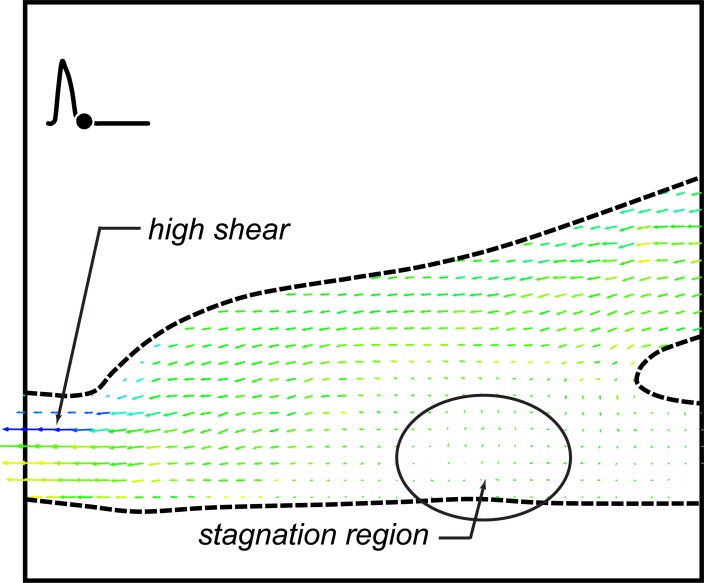
DPIV vector field for the straight end-to-side graft shortly after diastole for the excitation case. Observe that the stagnation region highlighted in Fig 7A remains throughout the cycle. Also note the high shear in the toe region even for low flow rates during diastole.

Fig 8 shows the straight end-to-side graft shortly after peak diastole. This vector field shows the full extent of the stagnation region. In this region, for the excitation case, there does not seem to be any flow throughout the cardiac cycle. In addition, it can be seen that in the toe region, the downstream attachment point of the cuff, the shear stress is high (blue) even for the low speed flows associated with diastole. One would expect this to occur in geometries where separated flow turns around a convex corner. Regions of perpetually high shear stress can cause cell damage as previously found [7].

### IV.2. Comparison of the two geometries with 10% retrograde flow (90–10 split)

#### IV.2a The rest case

**Fig 9:**
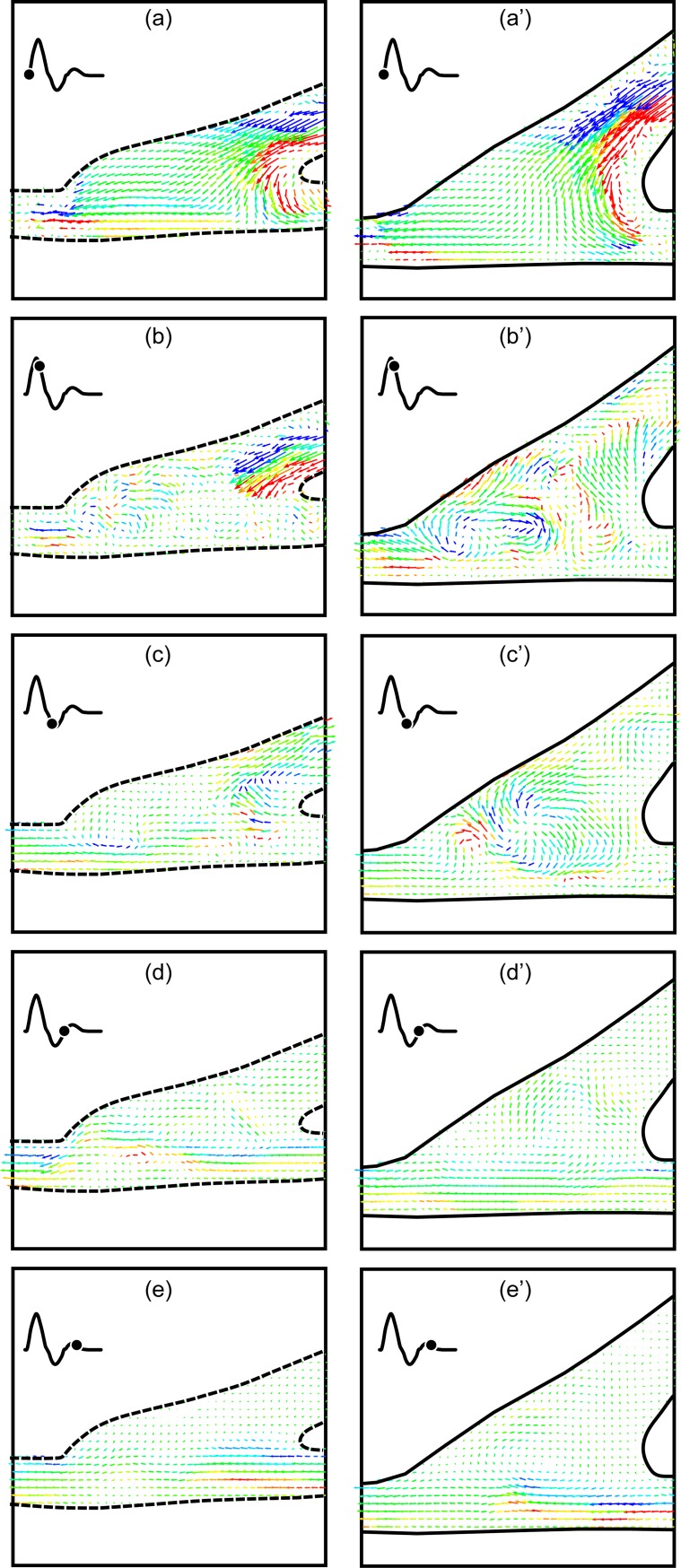
Side-by-side comparison of the two grafts for a rest cardiac cycle and 10% retrograde flow. Time between successive vector fields is 140 msec or ~17% of one cardiac cycle.

If there is leakage upstream through the occluded native artery, the flow becomes much more complex. This is shown in Fig 9 with two side-by-side five vector field sequences. The straight end-to-side sequence is again on the left and the contoured end-to-side is on the right. Unlike the sequences shown in Fig 4, every other vector field in one cardiac cycle is shown. The time between vector fields is 140 msec. Both sequences again begin at the start of the cardiac cycle.

It is abundantly clear that the overall flow is very different with and without retrograde flow, Figs 9 and 4, respectively. When retrograde flow is present, flow in the anastomosis for both geometries rapidly becomes irregular with swirling motions throughout. It is interesting to note that opening the proximal end of the native artery results in extended periods of flow along the bottom wall of that vessel. This can be seen in Fig 9D, 9D’, 9E and 9E’. This means that the problem of stagnation region along the floor of the anastomosis, *cf*. Fig 8, does not occur when there is retrograde flow.

It is also interesting that the flow wraps around the acute angle bend at the heel of the cuff. This is visible in Fig 9A and 9A’. Flow following a convex curve is known as the ‘Coanda effect’. Both the Coanda flow and flow along the bottom of the anastomosis result because of the change in pressure boundary condition on the proximal end of the native artery.

**Fig 10:**
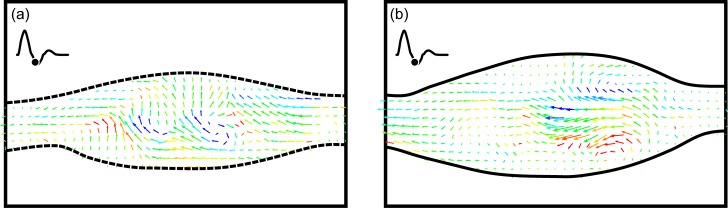
**Single DPIV vector fields showing bottom views of flow through the straight end-to-side (a) and contoured end-to-side (b).** The measurement plane is centered on the mid-plane of the native vessel. Images were shown at diastole.

Evidence of strong asymmetric three-dimensionality in the anastomosis can be seen in the bottom view measurements shown in Fig 10. As with other data, the straight end-to-side is shown next to the contoured end-to-side. The laser sheet is centered on the mid-plane of the native artery. Flow is right-to-left. Images were shown at diastole.

Asymmetry was present in both models for all four cases examined. One would expect this to be so. It also seems that the magnitude or intensity of the asymmetry is greater in the cases with retrograde flow. That is the swirl velocities are greater in the 90–10 split than the 100–0 cases. Because of the randomness of these circumferential swirls, the underlying endothelial cells may potentially experience quite complex hemodynamic loading. It is important to note that the measurements plane in Fig 10 is far from the bottom surface of the anastomosis; one cannot extrapolate the exact magnitude, direction and unsteadiness of the wall shear.

#### IV.2b The excitation case

**Fig 11:**
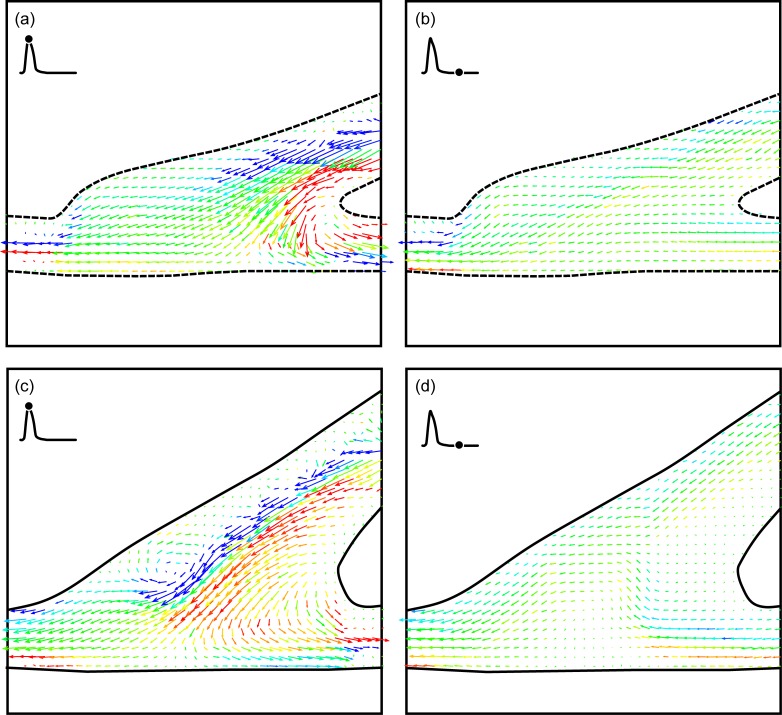
**DPIV vector fields for an excitation cardiac cycle and 10% retrograde flow for the straight end-to-side (a, b) and contoured end-to-side (c, d) grafts.** The time points shown are at peak systole (a, c) and diastole (b,d).

The fourth and final case examined was the 90–10 split with a cardiac cycle representing an excitation state. In many respects, this appears to be fluid dynamically the ‘cleanest’ of the four cases examined. For both graft models, roughly 75% of each cardiac cycle, flow through the anastomosis appears to be laminar and uniform. It is only at peak systole and diastole that there may be strong three-dimensional vortices as shown in Fig 11.

Because of the retrograde flow, extended stagnation regions do not develop across the anastomosis floor. In addition, reduction in speed through the bypass graft (some flow comes through the native artery) in the straight end-to-side seems to eliminate the recirculation region that appeared in the bulbous volume in the cuff. Consequently, for this specific flow condition, the advantages of the contoured end-to-side are not as evident.

## V. Discussion on the hemodynamics of arterial grafts

### V.1. On flow in healthy arteries

Consider first the differences between flows in the two geometries relative to the ‘base’ flow in a healthy artery. For a healthy artery, flow is everywhere along the axis of the vessel. For the idealized artery, flow is either forward, during systole, or stationary during diastole. In actuality, there is a slight amount of back flow during diastole because of elasticity of the vasculature.

Flow at arterial bifurcations becomes more complex [27]. The key complexities are the generation of axial swirl and potential for flow separation and reversal downstream of the branch. With the right combination of mitigating factors (*e*.*g*. hypertension, elevated cholesterol, smoking, genetic predisposition, *etc*.) these flow complexities can lead to occlusion through atherosclerosis [28, 29].

Vessel convergence, *i*.*e*. the bypass, is not a naturally occurring geometry in arteries. While veins converge into larger vessels, arteries typically do not. As such, bypass surgeries inherently introduce unnatural changes to the base flow that may, in turn, trigger intimal hyperplasia. The challenge for designing an optimal graft, then, is sculpting bypass geometries to minimize flow irregularities imposed on the native artery.

### V.2. On flow differences between the two geometries

With the prior discussion in mind, it is possible to consider differences in flows between the two bypass models being examined. The three primary geometric differences are:

1. the straight end-to-side has a bulbous cuff, particularly at the toe of the graft,
2. the angle between the bypass vessel and the native vessel is larger for the contoured end-to-side than for the straight end-to-side, and
3. the anastomosis volume for the contoured end-to-side is significantly larger than the straight end-to-side.

**Fig 12:**
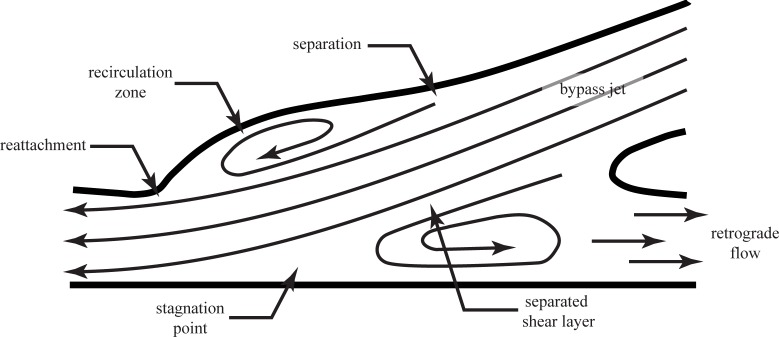
Schematic drawing of a graft model highlighting key flow features through the anastomosis at systole.

An important difference between graft models would seem to be the shape of the cuff. In the straight end-to-side graft, shown schematically in Fig 2, has a bulge in the toe region, analogous to the forehead of a bottlenose dolphin. As blood moves along the bypass into the cuff during peak systole, flow separates at the upstream end of this bulge forming a ‘recirculation zone’. At the point of separation, there is a sudden decrease in wall shear stress. At the toe of the cuff, the separated flow reattaches, resulting in a region of very high stress. These features are labeled as ‘separation’ and ‘reattachment’ in Fig 12. All along recirculation zone, strong shear layer vortices could form which will flow past the reattachment point and into the distal end of the native artery. This phenomenon is discussed in greater detail in the following paragraphs. By comparison, expansion in the contoured end-to-side cuff is more gradual and uniform. Flow is less likely to separate and produce strong and unsteady vortices.

The second hemodynamically important difference is the attachment angle of the grafts. The straight end-to-side model is attached at a smaller angle than the contoured end-to-side model. The argument in favor of a smaller attachment angle is the fact that the geometry more closely replicates a healthy straight artery. However, because it is not possible to have a truly 0° attachment angle, *i*.*e*. an end-to-end connection, the ‘bypass jet’ emanating from the graft will develop a ‘separated shear layer’, also shown in Fig 12. Shear layers, interfaces between high and low speed flow, are unstable to small disturbances which amplify and roll-up into a row of vortices. The size, strength and shedding frequency of these vortices depends on the velocity difference across the shear layer and the shear layer thickness. Since shear layers of this type tend to be very thin (the separation distance between the maximum speed and minimum speed flows is small), the vortices are usually small but highly energetic.

Shear layer vortices also travel with the flow, generally at a speed equal to the average of the maximum and minimum velocities on either side of the shear layer. As they are carried through the anastomosis, along the recirculation zone, they could significantly disturb the endothelium as they impinge on the lateral side (*i*.*e*. bottom) of the native vessel. As noted earlier, the same phenomena could occur in the bulbous cuff of the straight end-to-side graft.

Note that shear layer vortices are distinctly different from the large recirculation zone, shown in Fig 12. The recirculation zone is generally much larger than the shear layer vortices and remains nominally stationary. There may, however, be some meandering about a mean position as a result of unsteadiness associated with the formation and passage of shear layer vortices. When a leakage flow is present, the recirculation zone will be significantly altered, and may even cease to exist. Leakage out the occluded native artery (*i*.*e*. to the right), of course, creates an entirely different set of clinical problems.

The argument in favor of a larger attachment angle for the graft is that the size of the recirculation zone will be reduced. This means that the streamwise extent of the separated shear layer will be reduced. This in turn means that fewer, if any, shear layer vortices will be able to roll up before the flow impinges on the lateral native vessel wall. It also means that the length of native artery affected by the recirculation zone will be reduced; the region of highly unsteady, and potentially highly reversed flow is smaller with larger attachment angles.

There is one final flow feature which can play a major role in determining the relative efficacy of the two graft models. This is labeled ‘stagnation point’ in Fig 12. A stagnation point is a point where the flow velocity goes to zero. For the graft, the stagnation point defines the dividing point between the ‘bypass jet’ which reattaches and flows down the native artery, and the recirculation zone. Upstream of the stagnation point, blood will be transported upstream toward the occlusion against the natural flow direction. In the context of the endothelium, the stagnation point represents a point of zero shear.

In a healthy artery, virtually the entire endothelium will experience zero shear at some time in each cardiac cycle. This is quite natural and endothelial cells inherently adapt to this condition. What would be problematic is, if, throughout the cardiac cycle, one or more regions in an arterial system were in stagnation regions. Depending upon how the bypass graft is attached to the native artery, there is a danger of creating fixed stagnation points. In those regions, endothelial cells would perpetually not be subjected to shear stress. It is well known that endothelial cells respond very differently under zero shear conditions [30].

In addition to the regions of zero shear stress, a fixed stagnation point on the native artery implies a correspondingly fixed recirculation zone. This is highly undesirable because of the very slow transport rates across the recirculation zone. Fluid in a recirculation zone, by definition, recirculates. This means that transport of fresh nutrients and removal of waste to/from endothelial cells within the recirculation zone will be significantly diminished. This is obviously a highly undesirable condition. Even if the cells in that region were not negatively impacted by low shear, they would be damaged due to the buildup of waste and the lack of fresh nutrients. In this context, some degree of turbulence that disrupts a recirculation zone could be positive.

## VI. Summary and conclusions

Spatially and temporally resolved measurements of flow in a straight end-to-side and contoured end-to-side model were made using Digital Particle Image Velocimetry. Four different cases were examined for each model, including two different cardiac cycles (rest and excitation) and two upstream conditions for the native artery (100-0/no retrograde flow and 90-10/retrograde flow [distal:proximal]). Measurements were made from the side (in the symmetry plane of the graft model) and from below. Summary observations and conclusions made from these measurements include:

1. For 3 of the 4 cases studied, flow in the contoured end-to-side model is more laminar and more uniform than the straight end-to-side design. (In the 90–10 flow split under excitation, flow in both grafts appears comparable.)
2. At the same time (as a result), the magnitude of shear stress fluctuations in the contoured end-to-side model is lower than in the straight end-to-side.
3. The straight end-to-side model develops stagnation regions along the anastomosis floor which may persist for most of the cardiac cycle. This means that there may be a lack of turnover of fresh oxygenated blood to the underlying endothelium.
4. Flow in the straight end-to-side model separates entering the anastomosis because of the bulge in the toe region. This results in a recirculation region with very low shear and may lead to reduced transport of oxygenated blood.
5. Flow reattaches at the toe causing very high shear stress, which could cause cell damage.

In addition, our results show that the design of an optimal shape for a bypass graft depends strongly on the flow waveform and the presence of retrograde flow through the proximal end of the native artery.
